# The burden of injuries in Ethiopia from 1990-2017: evidence from the global burden of disease study

**DOI:** 10.1186/s40621-020-00292-9

**Published:** 2020-12-21

**Authors:** Solomon Ali, Zelalem Destaw, Awoke Misganaw, Asnake Worku, Legesse Negash, Abebe Bekele, Ababi Zergaw, Ally Walker, Chris Odell, Mohsen Naghavi, Ebba Abate, Alemnesh H. Mirkuzie

**Affiliations:** 1 Ethiopian Public Health Institute (EPHI), Addis Ababa, Ethiopia; 2 Saint Paul’s Hospital Millennium Medical College, Addis Ababa, Ethiopia; 3 Institute for Health Metrics and Evaluation, Department of Health Metrics Science, University of Washington, Seattle, USA; 4 University of Bergen, Bergen, Norway

**Keywords:** Road injury, Falls, Self-harm, Violence, DALYs, Deaths, EPHI, Ethiopia

## Abstract

**Background:**

Mortality caused by injuries is increasing and becoming a significant global public health concern. Limited evidence from Ethiopia on road traffic, unintentional and intentional injuries indicate the potential public health impact of problems resulting from such injuries. However, there is a significant evidence gap about the actual national burden of all injuries in Ethiopia. This data base study aimed to reveal the national burden of different injuries in Ethiopia.

**Methodology:**

Data for this study were extracted from the estimates of the Global Burden of Diseases (GBD) 2017 study. Estimates of metrics such as Disability-Adjusted Life Years (DALYs), death rates, incidence, and prevalence were extracted. The metrics were then examined at different injury types, socio-demographic categories such as age groups and sex. Trends of the metrics were also explored for these categories across years from 2007 to 2017. The DALYs and deaths due to injuries in Ethiopia were also compared with other East African countries (specifically Kenya, Tanzania, Uganda, and Zambia) in order to evaluate regional differences across years, by sex and by different injury types such as transport injuries, unintentional injuries, self-harm and interpersonal violence.

**Results:**

The age-standardized injury death rate has decreased to 69.4; 95% UI: (63.0–76.9) from 90.11; 95% UI: (82.41–97.73) in 2017 as compared with 2007. Road injury, falls, self-harm and interpersonal violence were the leading causes of mortality from injuries occurring in 2017. The age-standardized injury DALYs rate has decreased to 3328.2; 95% UI: (2981.7-3707.8) from 4265.55; 95% UI: (3898.11–4673.64) in 2017 as compared with 2007. The number of deaths resulting from injuries in 2017 was highest for males, children under 5 years, people aged 15–24.

**Conclusion:**

The current age-standardized death rate and DALYs from injuries is high and the observed annual reduction is not satisfactory. There is a difference in gender and age regarding the number of deaths resulting from injuries. The data indicates that the current national efforts to address the public health impact of injuries in Ethiopia are not sufficient enough to bring a marked reduction. As a result, a more holistic approach to address all injuries is recommended in Ethiopia.

**Supplementary Information:**

The online version contains supplementary material available at 10.1186/s40621-020-00292-9.

## Introduction

Injury is defined as the physical damage resulting from exposure to sudden energy that exceeds the threshold limit of human physiological tolerance (Baker et al. 1992). Transport injuries, unintentional injuries (drowning, falls, exposure to mechanical forces, fire, heat and hot substances) and intentional injuries (interpersonal violence and self-harm) cause approximately 3.5 million deaths globally (Injury facts international, 2016).

Injuries in general, and road traffic injuries in particular, are a growing national concern in Ethiopia. In the year 2014/15 alone, there were 15,086 road traffic crashes which caused an estimated economic loss of 7.3 million USD (Woldegebriel et al. 2019). In the same year, the fatality rate compiled from Addis Ababa-Adama-Hawassa city road data was 156/10,000 vehicles (Abegaz et al. 2014). One year later, (the year 2016) the road traffic mortality rate per 100,000 population for Ethiopia was 26.7 (WHO global health data repository and road traffic death data by country, 2016).

The observed road traffic crashes and resulting mortalities are high despite limited main road coverage and number of cars driven in the country. Ethiopia is a country with total surface area of 1,104,300 km^2^ (The world fact book, 2020). Despite this, the 2015 main road coverage of the country was only 100,000 km. Recently, the Ethiopian Road Authority has increased this coverage to 200,000 km as part of Ethiopia’s national Growth and Transformation Plan II (GTPII) (GTP II midterm evaluation 2018). The number of cars driven in Ethiopia was estimated to be 831,265 in 2017, 62% of which are driven in Addis Ababa, the capital city of Ethiopia (Ethiopian business alert 2017).

Ethiopia has been legislating different rules and regulations targeted to manage traffic flow with the ultimate goal of reducing traffic accidents. The driver’s qualification certification license proclamation number 1074/2018, transport restructuring proclamation number 468/2005, regulation on vehicles identification, inspection and registration fees council of Ministers regulation 206/2011, National Road Traffic ‘Safety Council Establishment Regulation no. 205/2011 and Motor Vehicles and Trailers Identification, Inspection and Registration (Amendment) Regulations 74/2001 are the main road traffic rules and regulations (Ethiopian Transport and Communication laws 2011).

In response to these legislative efforts, the National Road Traffic Safety Council (NRTSC) was established (Ethiopian national road safety management frame work 2019), a national strategy for road safety was developed, targets to reduce road traffic deaths were set, and a road safety management framework was created (Federal Negarit Gazeta 2011). Furthermore, there is a daily, monthly and annual traffic injury report which is transmitted to the public through different mass media outlets and communication platforms in order to raise public awareness about road safety.

Injuries from drowning, falls, fire, heat and hot substances, and mechanical forces are neglected in Ethiopia. Data about the burden of such injuries are very limited. There are few published studies that indicate the magnitude of deaths resulting from injuries. Recent 2020 evidence from Addis Ababa Mortality Surveillance System using verbal autopsy indicates that injuries contributed for 7% of the total deaths (Anteneh and Endris, 2020). According to data compiled from six Health and Demographic Surveillance Sites (HDSS) at universities in Jimma, Arba Minch, Butajira, Dabat, Kilet Awulalo and Kersa, of the total 9719 deaths that were registered between 2009 and 2013, 623 (6.4%) resulted from injuries (Gelaye et al., 2018). Drowning caused 126 (21.8%), falls caused 113 (18.1%) and road traffic crashes caused 112 (18.0%) injury related deaths (Gelaye et al., 2018). The findings of this study outlined the growing public health challenges associated with unintentional injuries that demand an immediate response in the country. According to the 2016 Ethiopian Demographic and Health Survey, the magnitude of unintentional injuries and fatalities were 163 (95% CI: 136–195) and 37 (95% CI: 25–54) per 100,000 population respectively (Abegaz and Gebremedhin, 2019). Additional evidence from a health institution based research study conducted among 384 trauma victims in Wolaita zone hospitals has listed road traffic crashes (62.5%), falls (20.8%) and drowning (9.6%) as the leading causes of trauma (Hailemichael et al. 2015). One systematic review has indicated the pooled road traffic injuries among trauma victims in Ethiopia is 31.5% (Endalamaw et al., 2019).

To the best of our knowledge, there are no clear guidelines, initiatives, strategies, targets and research directions in place that match with the growing problem of injuries resulting from falls, drowning, mechanical forces, fire, heat and hot substances in Ethiopia. Furthermore, intentional injuries such as self-harm and interpersonal violence have not received enough attention. As a result, the actual burden and trend of such injuries in Ethiopia remains unclear. The limited evidence on the burden of such injuries either come from health facilities based or are fragmented (Abegaz and Gebremedhin, 2019, Alemu Gelaye et al., 2018, Hailemichael et al. 2015).

Hence, this article aims to fill evidence gaps on injury burden to guide public health policy in Ethiopia using the 2017 Global Burden of Diseases, Injuries, and Risk Factors Study (GBD) data. This article estimates injury incidence, prevalence, mortality, and disability-adjusted life years (DALYs) resulting from unintentional and intentional injuries.

## Methodology

### Settings

Ethiopia is the second most populous country in Africa. According to the Central Statistics Agency (CSA) 2017 projection, Ethiopia has an estimated population of 94,351,001 people with 80% of this population living in rural settings (Central statistics agency population projection for Ethiopia between 2007–2037, 2020). The country is one of the fastest growing economies in Africa (World economy forum 2018). The Ethiopian Public Health Institute (EPHI), a research arm for the Federal Ministry of Health, is collaborating with the Institute for Health Metrics and Evaluation (IHME) at the University of Washington to produce valid and reliable national and sub-national disease burden estimates including injury. The institute has established the National Data Management Center (NDMC) for health, which is responsible for addressing the information revolution agenda of the Health Minister and generating strong evidence for policy and high-level decisions.

### Data processing approach summary

#### GBD methods and data source

The Global Burden of Diseases, Injuries and Risk factor study (GBD) is a comprehensive global study updated annually since 2015. It allows comparisons of the magnitude of diseases, injuries, and risk factors across age groups, sexes, countries, regions, and time. Information generated from the GBD study can be used to monitor health progress, make informed decisions and indicate policy directions. It also helps to understand the leading causes of health loss that could potentially be averted (GBD 2017a-GBD 2017f).

The causes of mortality and morbidity are structured using a four-level classification hierarchy, based on International Classification of Diseases (ICD), and used for modeling to produce results that are mutually exclusive and collectively exhaustive (GBD 2017b, GBD 2017c). GBD 2017 classifies 195 countries into 21 regions based on epidemiological similarities and geographical proximities, seven super-regions, and produces subnational estimates for few countries.

Cause-specific mortality was estimated using Cause of Death Ensemble model (CODEm). CODEm produces several plausible combinations of covariates using a covariate selection algorithm. The combinations of covariates are then analyzed using mixed effects linear models and spatiotemporal Gaussian process regression models for cause fractions and death rates (GBD 2017b, GBD 2017e).

The GBD 2017 calculated its own population and fertility estimates (GBD 2017f) and used the GBD world population age standard to calculate age-standardized rates for cause-specific deaths across all fatal and non-fatal estimates (GBD 2017b, GBD 2017e, GBD 2017f). GBD 2017 creates the estimates to provide age-standardized numbers and rates of each metrics along with 95% uncertainty intervals (UI). Uncertainty intervals are a range of values that are likely to include the correct estimate of age-standardized metrics for a given cause (GBD 2017a).Accordingly, the extracted results for this study are presented with UI to show the precision of a particular metrics or estimate. The GBD 2017 study provides estimates of Years of Life Lost (YLLs), Disability-Adjusted Life Years (DALYs), death rates, incidence, and prevalence. We have extracted the metrics from GBD 2017 data to estimate the burden of injuries in Ethiopia. GBD has calculated years of life lost (YLL) from the sum of each death multiplied by the standard life expectancy at each age (GBD 2017b). Years lived with disability (YLD) are calculated from the prevalence of each disease sequela multiplied by the disability weight. Disability-adjusted life years (DALY), are calculated as the sum of YLL + YLD by cause. Additional detail about the techniques of estimation for these metrics is available in the supplemental materials in the GBD 2017 DALYs and HALE capstone (GBD 2017b) and the GBD 2017 Disease and Injury Incidence and Prevalence capstone (GBD 2017d).

The GBD injury specific estimates for Ethiopia for 2017 were derived from data obtained from different sources, such as Addis Ababa Mortality Surveillance Program, Ethiopia Demographic Surveillance Verbal Autopsy (please see additional file 1), which were collected using verbal autopsy or survey methods, among other publications. Thus, data for this study was extracted from the estimates of the GBD 2017 from IHME’s Global Health Data Exchange (http://ghdx.healthdata.org/).

#### GBD Ethiopian data extraction and analysis

The statistics about the overall injury was summarized from the available GBD data that covers information about animal contact, conflict and terrorism, drowning, environmental heat and cold exposure, executions and police conflict, exposure to forces of nature, exposure to mechanical forces, falls, fire, heat, and hot substances, foreign body, interpersonal violence, other exposure to mechanical forces, other unintentional injuries, physical violence by firearm, physical violence by other means, physical violence by sharp object, poisoning by gas, poisonings, self-harm, self-harm and interpersonal violence, transport injuries, unintentional firearm injuries, unintentional injuries, and venomous animal contact. Detailed sources of data for each specific injury type can be referred to in additional file 1.

The estimates of the metrics such as DALYs, death rates, incidence, and prevalence were extracted covering the periods 1990 to 2017. The metrics were then examined and summary statistics of the numbers and age-standardized rates of the metrics by different socio-demographic categories such as age groups and sex, and by different injury types were presented. Trends of the metrics were also explored for these categories across years 1990 to 2017. Number of deaths due to injuries was examined and summary statistics are provided by categories such as all ages and sexes for 2017. Uncertainty intervals for each summary statistics are presented along with the numbers and rates. The GBD study provides estimates disaggregated at several age groups. For convenience of presenting the analysis results, age groups were categorized into “under-5” years, “5–14” years, “15–24” years, “25–34” years, “35–44” years, “45–54” years, “55–64” years, “65–74” years, “75+” years and examined against the total death due to injuries and specifically, against deaths due to transport injuries, unintentional injuries, self-harm and interpersonal violence. The differences were compared by sex and by years.

Similarly, the DALYs and deaths due to injuries in Ethiopia were also compared with other East African countries (specifically Kenya, Tanzania, Uganda and Zambia) in order to evaluate intra-regional differences across years, by sex and by different injury types such as transport injuries, unintentional injuries, self-harm and interpersonal violence.

## Results

### Burden of injuries overview

**Fig. 1:**
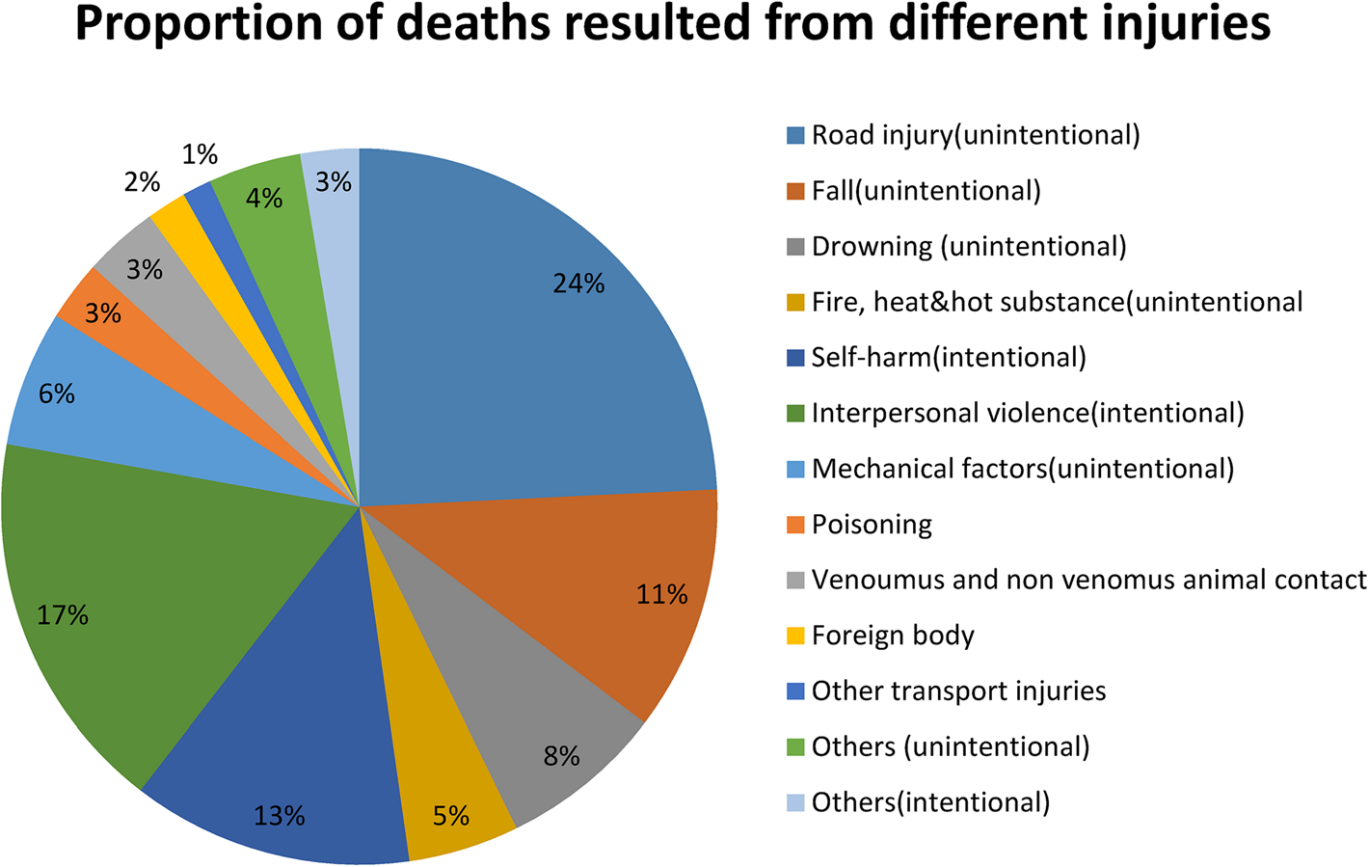
Percentage compositions of deaths from different injuries in Ethiopia, GBD 2017. Others-unintentional injuries include injuries resulting from environmental heat, exposure to force of nature and unspecified; Others-intentional injuries includes conflict and terrorism, execution and police conflict

The total number of deaths, DALYs and prevalent cases from injuries in 2017 were 41,834 (95% UI: 37,703-46,584), 2,845,543 (95% UI: 2,551,985-3,181,088) and 13,886,462(95% UI:12,933,583-14,874,465) respectively. From the observed annual deaths, 65% were from road injuries, interpersonal violence, self harm and fall injuries (Fig. 1).

### Burden of injuries by age and sex

**Fig. 2:**
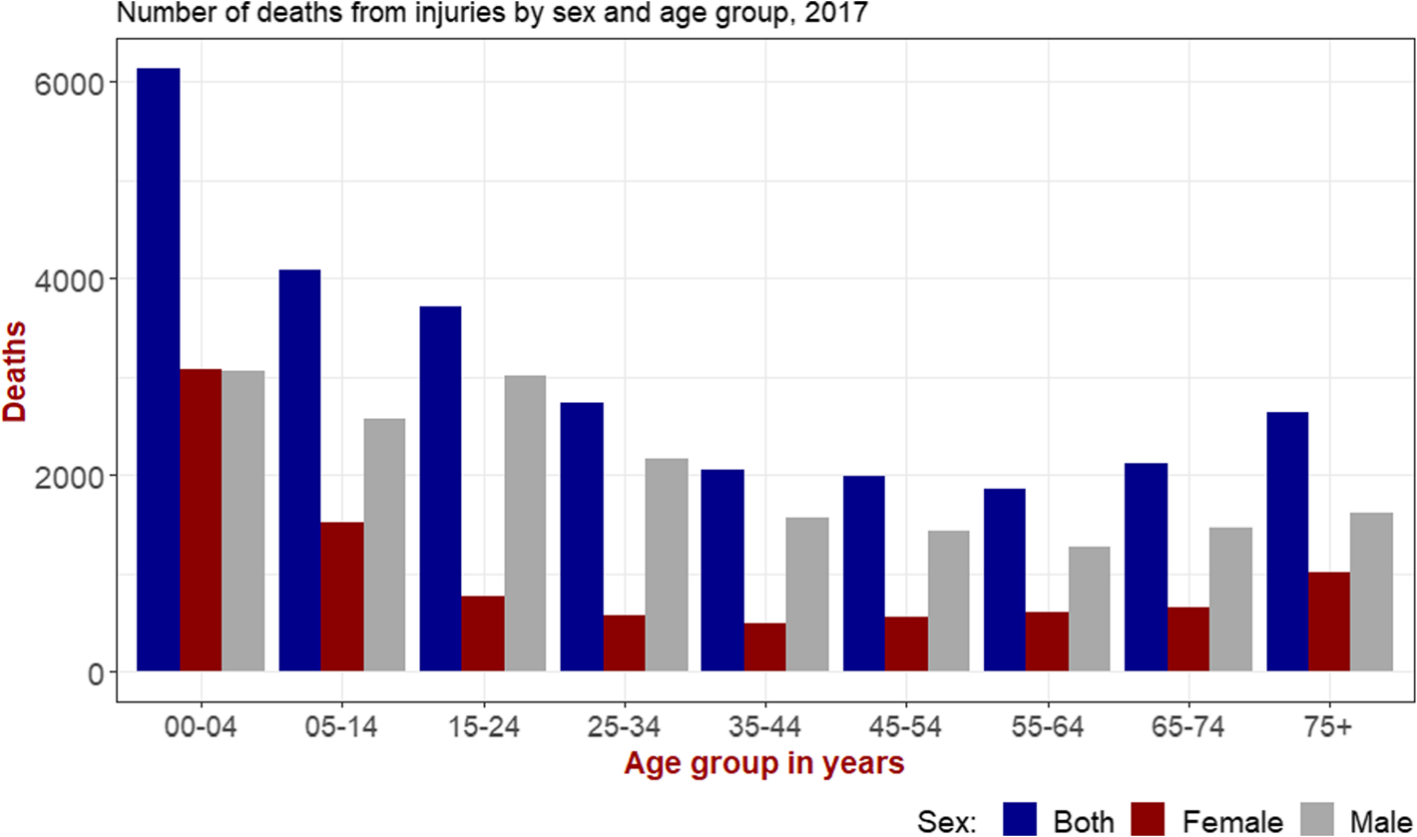
Number of deaths due to injuries by age and sex, GBD 2017

In 2017 the number of deaths resulting from injuries among males (28,565, 95% UI: 25,100-32,738) was more than two times higher than females (13,269, 95% UI: 11,661–14, 806) (Fig. 2: Additional file 2). This difference was consistent with the age-standardized rate and throughout the 28-year mortality trend observed. More than half (52%) of all deaths due to injuries have occurred among ages under 35 (Fig. 2). The number of deaths is highest among the under-5 and 15–24 age groups (Fig. 2). Stratification of the number of deaths by injury types in 2007 and 2017 has indicated a consistently higher number of deaths among males though this difference was not seen in children under 5 (Additional file 2, Additional file 3).

### Age-standardized mortality

The overall 2017 age-standardized mortality from all injuries was 69.4 (95% UI: 63.0–76.9) per 100,000 population. In the same year the age-standardized mortality from road injury, falls, self harm, interpersonal violence and drowning were 15.4 (95% UI: 14.0–16.9), 13.1 (95%UI:11.5–15.0), 9.6 (95%UI:8.3–11.2), 9.5 (95% UI:8.1–12.6) and 3.1 (95%UI:2.8–3.5) respectively.

### Comparison of age-standardized mortality in the last 10 years

We have assessed the data sources used to estimate the burden of injuries in Ethiopia. Most of the data used by GBD are generated in the last 10 years (2007–2017). Accordingly, we have compared the 2017 age-standardized mortality estimate with 2007 to get better picture of injuries in Ethiopia.

The age-standardized death rate per 100,000 population has decreased to 69.4 (95% UI: 63.0–76.9) in the year 2017 from 90.11 (95% UI: 82.4–97.7) in 2007. In 2017, there was a 4.6% reduction in the number of total deaths from all causes of injuries as compared with 2007 (Table 1). In a stratified analysis, the number of deaths resulting from intentional injuries (self-harm and interpersonal violence) has increased in 2017 by 6.6% as compared with 2007 (Table 1).

**Table 1:** Total number of deaths from injuries, age-standardized mortality rates and percentage of change in Ethiopia from 2007-2017, GBD 2017

Types of Injury	Number of deaths				% Change	Age standardized death rate per 100,000 population				% Change
	2007		2017			2007		2017		
	Number	95% UI	Number	95% UI		Rate	95% UI	Rate	95%UI	
Injury (all types)	43,838	(40,002-47,711	41,834	37,703-46,584	−4.6	90.11	82.41–97.73	69.44	62.99–76.9	− 22.93
Transport injuries	11,353	10,311-12,357	10,266	9335-11,312	−9.6	22.25	20.57–23.95	16.02	14.51–17.47	−28.0
Unintentional injury	19,836	17,421-22,301	18,084	15,536-20,989	−8.83	41.84	36.5–47.78	32.82	28.29–37.64	−21.6
Self-harm and violence	12,649	11,520-15,441	13,484	11,920-17,080	6.6	25.96	23.65–29.37	20.6	18.28–24.56	−20.6

### Incidence, prevalence and DALY of injuries in Ethiopia 2017

From transport injuries, the highest incidence, prevalence and mortality were due to road injuries (Table 2). From unintentional injuries, the age-standardized rate of incidence, prevalence, DALYs and mortality resulting from falls, drowning, and fire, heat and hot substances injuries were highest. Likewise among intentional injuries, interpersonal violence injuries caused the highest age-standardized incidence, prevalence and DALYs rate per 100,000 population (Table 2).

**Table 2:** The 2017 age-standardized Incidence, prevalence and DALY rates estimates by causes of injuries per 100,000 population, GBD 2017

Cause of injury	^a^Incidence (95%UI)	^a^Prevalence (95% UI)	^a^DALY (95%UI)
All causes injuries	6328 (5973–6717)	19,425.0 (18,197.0-20,857.0)	3328.2 (2981.7-3707.8)
**Transport injuries**	**477.7 (417.1–545.2)**	**1620 (1518-1743)**	**706.9 (642–778.3)**
Road injuries	390.0 (330.8–457.6)	1203.6 (1118.8–1297.2)	642.2 (584.7–706.8)
Other transport injuries	87.5 (73.6–105.4)	416.4 (353.8–476.6)	64.7 (51.1–80.1)
^**b**^**Unintentional injuries**	**5094.5 (4743.0-5451.6)**	**10,248.0 (9619.5-11,005.9)**	**1528.5 (1330–1764.1)**
Falls	1860.7 (1614.4-2166.6)	3908.5 (3456.7–4410.6)	465.5 (387.5–551.0)
Drowning	4.0 (3.4–4.6)	22.7 (20.0–25.9)	163.4 (141.1–191.1)
Fire heat and hot substances	131.1 (108.9–157.6)	1322.9 (1146.6-1511.5)	204.0 (168.2–252.1)
Poisoning	65.4 (50.7–83.6)	69.9 (58.3–82.6)	69.6 (53.7–86.2)
Exposure to mechanical forces	1105.2 (954.3–1279.2)	2642.5 (2322.8-3041.5)	241.7 (195.5–318.1)
Adverse effects of medical treatment	123.8 (103.5–145.7)	9.5 (7.3–11.9)	72.6 (47.5–105.7)
Animal contact	943.7 (820.3–1087.4)	730.4 (649.3–823.9)	100.8 (72.8–131.3)
Foreign body	427.8 (370.9–495.9)	393.3 (247.5–345.2)	56.4 (48.3–65.7)
Others	322.7 (247.6–383.5)	824 (713.6–967.07)	76.5 (63.1–93.2)
^**c**^**Intentional injuries**	**755.8 (675.3–839.6)**	**7567.4 (6522.1-8815.3)**	**1092.8 (967.9–1290.8)**
Self-harm	30.5 (25.4–36.2)	68.8 (60.1–80.2)	309.3 (268.3–359.4)
Interpersonal violence	464.4 (397.3–538.4)	4189.3 (3756.6-4691.0)	502.5 (434.2–670.6)

^a^ age-standardized rate per 100,000 population; ^b^Transport injury is not included here; ^c^Conflict and terrorism, and execution and police conflict are not included

### Injury mortalities across years in Ethiopia

**Fig. 3:**
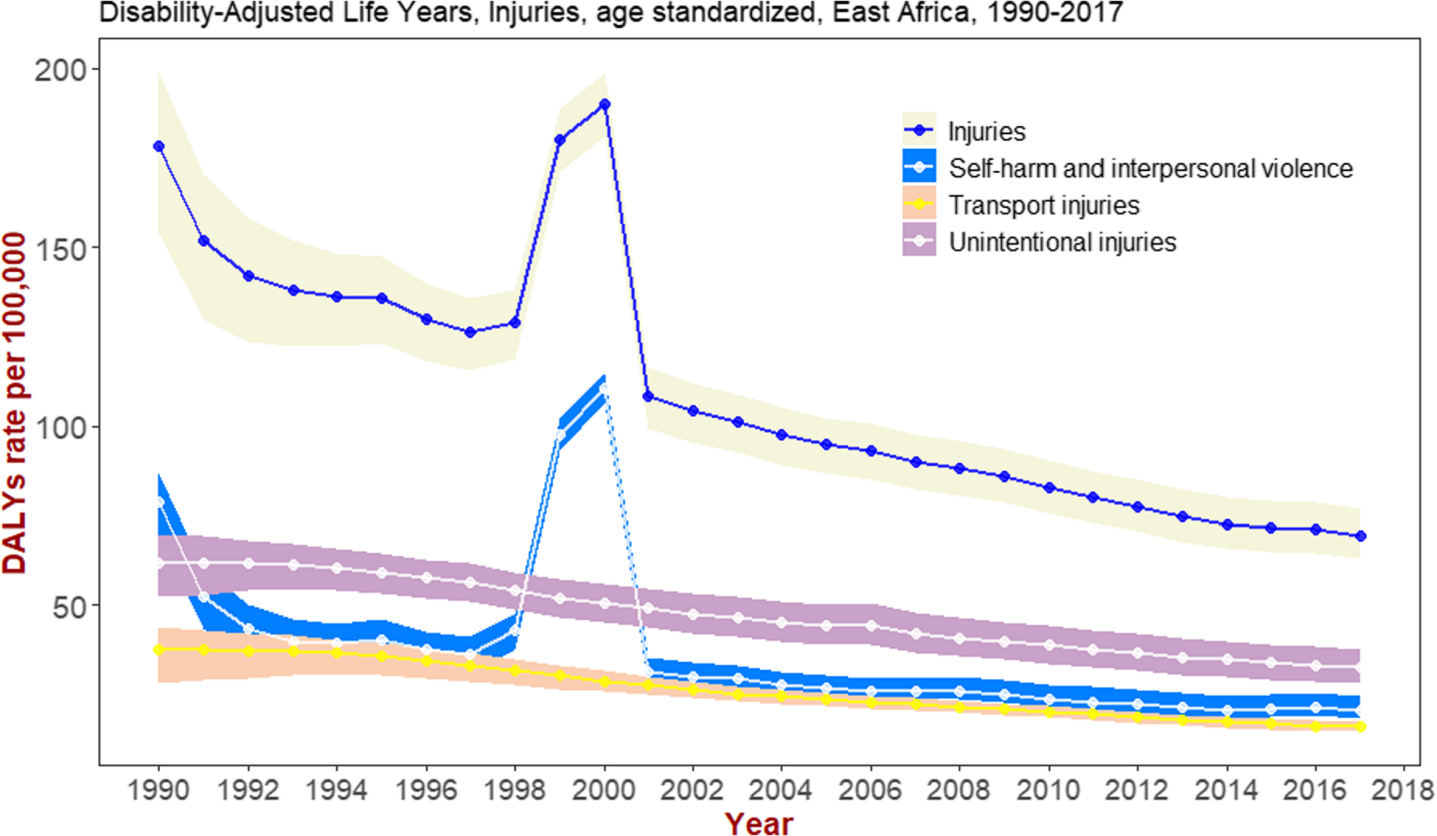
Deaths resulting from different types of injuries across years in Ethiopia, GBD 2017. Unintentional injury includes injury due to mechanical factors, falls, drowning, exposure to fire heat and hot substance

The number of deaths resulting from injuries in 1999 and 2000 were 89,023 (95% UI: 83514–93,915) and 98,247 (95% UI: 93,197-102,943) respectively (Fig. 3). These estimates were higher than estimates for all other years. Furthermore, two-thirds of these deaths seen in the 2 years resulted from violence injuries.

### Disability-adjusted life years (DALYs)

The number of DALYs resulting from all cause injuries have shown an overall decrease of 6.1% in 2017 as compared with 2007. In a stratified analysis, the number of DALYs from intentional injury that included self-harm and interpersonal violence has shown a 2.7% increase in 2017 as compared with 2007. The age-standardized DALYs rate per 100,000 population has shown 22% reduction in 2017 as compared with 2007. The highest reduction of age-standardized DALYs rate (28.6%) was observed from road injuries in 2017 as compared with 2007 (Table 3).

**Table 3:** Total disability-adjusted life years (DALYs) and age-standardized DALYs for injuries that cause death in 2007 and 2017 with change in percent, GBD study, Ethiopia, 2017

Types of Injury	Number of DALYs				% Change	Age-standardized DALYs rate per 100,000 population				% Change
	2007		2017			2007		2017		
	Number	95% UI	Number	95% UI		Rate	95% UI	Rate	95%UI	
Injury	3,031,560	2,740,292-3,325,418	2,845,542	2,551,985-3,381,088	−6.1	4265.5	3898.1–4673.64	3328.2	2981.6-3707.84	−21.9
Unintentional	1,454,424.63	1,272,438.83-1,626,493.34	1329,647.82	1,158,929.53-1,537,176.3	−8.6	1905.04	1682.37–2.126.81	1528.47	1329.96-1764.15	−19.8
Self-harm and violence	881,846.74	781,591.88-1,068,743.16	905,853.98	796,829.98-1,115,344.27	2.7	1369.7	1227.37-1605.11	1092.81	967.94–1290.83	−20.2
Road injuries	695,288.56	621,778.21-757,443.89	610,040.16	551,153,99-675,328.12	−12.2	990.81	909–1068.25	706.89	642.05–778.29	−28.6

### Burden of injuries in selected east African countries

**Fig. 4:**
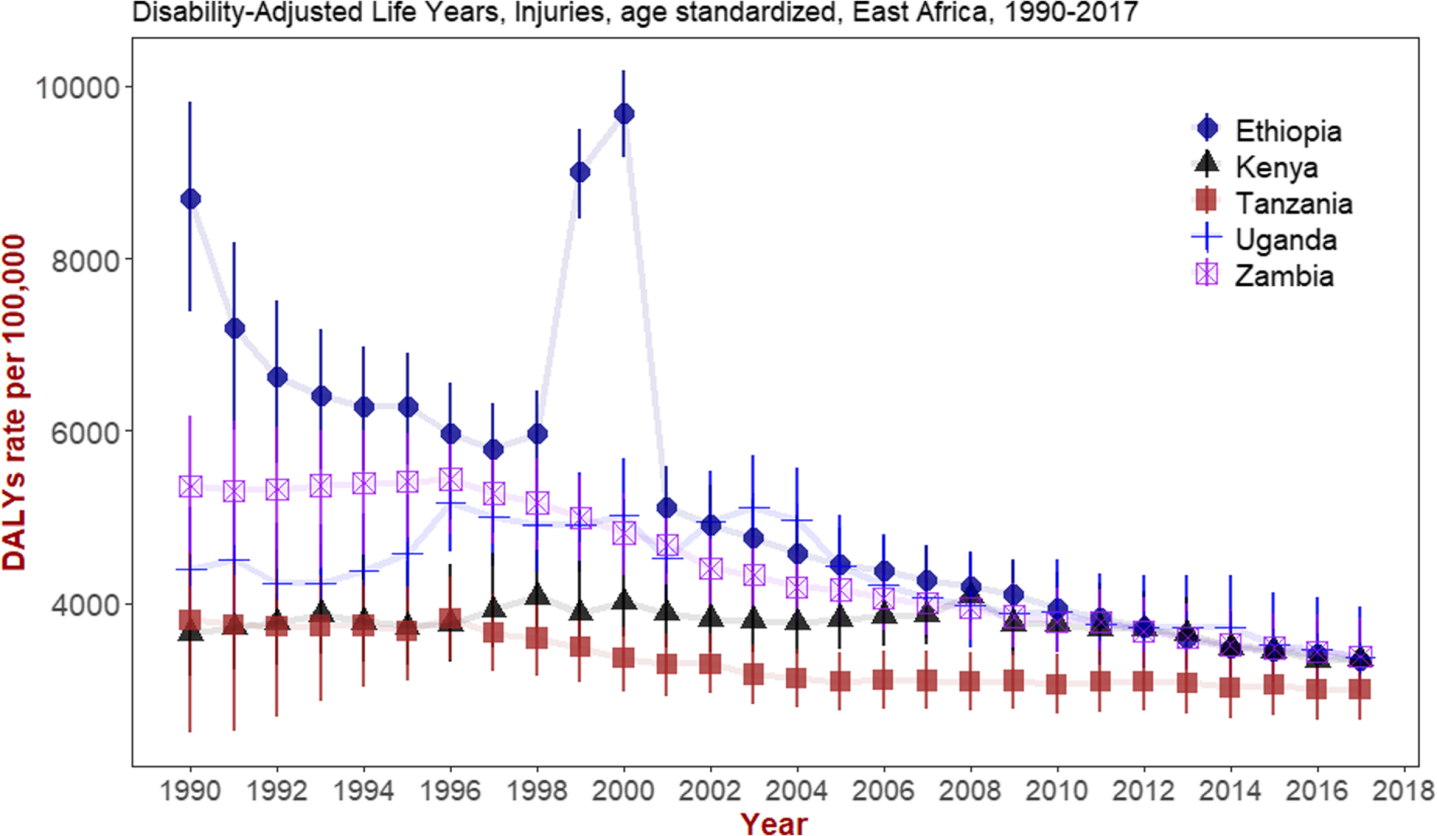
Trend of age-standardized DALYs resulting from injuries in Ethiopia, Kenya, Tanzania, Uganda and Zambia, GBD 1990–2017

As shown in Fig. 4, the age-standardized rate of DALYs in Ethiopia has steadily declined between 1990 and 2017. There was a peak between 1998 and 2001, and since then it has shown gradual and steady reduction.

The trend of DALYs in Kenya and Uganda seems consistent across years. In comparison, the magnitude of DALYs observed in Ethiopia was higher than that observed in Kenya, Tanzania, Uganda and Zambia until 2009. Afterwards, the observed burden of DALYs in Ethiopia has become consistent with these countries except Tanzania (Fig. 4). The DALYs and deaths stratified by type of injuries and by gender among East African countries is consistently higher for males than females (Additional file 3).

## Discussion

In 2017, a total of 41,834 peoples have died of injuries in Ethiopia. Nearly three-fourth of these deaths was resulted from road injury, inter-personal violence, self harm, fall and drowning.. These findings are different from Health and Demographic Surveillance System (HDSS) sites study which has reported drowning (21.8%), fall (18.1%), transport injury (18%), self harm (10.3) and assault (10.4%) as a leading causes of injury related deaths. This difference could be justified by the difference in data source. As indicated nearly 80% of HDSS sites data arefrom from rural settings. Such data better represents rural settings than Ethiopia (Gelaye et al., 2018).

The distribution of the number of deaths from injuries by age and sex has indicated that men, children under 5 (both genders) and young adults (15–24 years) are the most affected groups with injuries. These findings are consistent with a report from data generated using Ethiopian Demographic and Health survey (EDHS) and HDSS sites in Ethiopia (Abegaz and Gebremedhin, 2019: Gelaye et al. 2018). Further analysis of the GBD 2017 data indicates that most of the injury related deaths for children under five (76%) were caused by drowning, falls and mechanical forces while only 14.4% deaths were attributed to road injuries. Among young adults aged 15–24 years, road injuries accounted for 31.2% of the total deaths while falls, drowning and mechanical forces accounting for 21.3% of the total observed deaths in this age group.

The order of age standardized death rate per 100, 000 population resulted from injuries in 2017 is different from the observed order by number of deaths. As a result, road injury, falls, self harm, interpersonal violence and drowning were the leading causes of age standardized injury deaths in Ethiopia.

The age-standardized death rate resulting from road injuries in 2017 was 15.4 per 100,000 population. This annual death rate estimate is very concerning due to the fact that the number of vehicles in Ethiopia is less than one million (Ethiopian business alert 2017). In comparison, the observed death rate due to road injuries in Ethiopia is consistent with the pooled estimate (16.6) of 15 African countries that included Ethiopia (Adeloy et al. 2016). Several factors might contribute to this high death rate due to road injuries in Ethiopia, including limitations in capacity and resources to implement and enforce available legislation, policies and guidelines designed to ensure road safety in Ethiopia. The country has policies and directives on speed limits, drinking and driving, motorcycle helmet use, wearing seat belt and mobile phone use while driving (Abegaz et al. 2014, Federal Negarit Gazeta number 8, 2017). We speculate that drivers’ behavior and drivers’ limited competency may result from substandard training, and a lack of national standards for imported cars while inadequacy of post-crash care might also contribute to the observed high death rate.

The burden of falls, drowning and mechanical forces remains unknown in Ethiopia. The present study has provided evidence on the age-standardized death rate due to falls and drowning, which were 13.13 and 3.11 per 100,000 population respectively in 2017. We found no other evidence with which to compare the present findings from Ethiopia. Available literature about injuries were from the health facilities and mainly focused on characterizing injury victims, the severity of the disease and the injury outcomes (Hailemichael et al. 2015, Seid et al. 2015, Gebresenbet and Aliyu, 2019).

Falls and drowning are highly preventable public health challenges and yet poor attention is given to target preventive strategies in Ethiopia. There is no policy or response in place that matches with the burden of deaths and morbidities from falls and drowning. The contributing factors for the observed deaths from falls and drowning is not well studied. However, we speculate that the widely distributed water wells, small ponds quarries and ditches near residential areas in rural and in semi-urban areas, and ever-increasing construction in urban settings might increase the risk of drowning and/or falls. Furthermore, lack of awareness about construction safety management and absence of an established system to ensure safety for the construction workers and the surrounding community might play a significant role for the observed number of deaths.

Intentional injuries, mainly self-harm and interpersonal violence, are also a significant public health problem in Ethiopia. The age-standardized death rate resulting from intentional injuries was 20.6 per 100,000 population in 2017. This estimate is almost 30% of the total injury related deaths observed. The age-standardized death rate from self-harm was 9.63 and that of interpersonal violence was 9.54 per 100,000 populations. The observed death rate due to interpersonal violence might be explained by the mass uprising, unrest and violence that happened in Ethiopia during 2015/2016 which led to political instability and later forced the ruling party to undergo change and political reform.

Self-harm is a neglected but important health problem in Ethiopia. Most available evidence on self-harm is focused on suicidal behavior, ideation and attempts mostly among psychiatric patients (Jordans et al. 2018). The latest 2017 GBD estimate of deaths due to self-harm injuries clearly demands immediate response and highlights the need to consider self-harm as one of the important public health conditions.

Our study has also assessed the trend of age standardized deaths across years. The age-standardized death rate resulting from road injury has decreased by 28.6% in the past 10 years. However, the observed annual reduction rate is not sufficient enough for Ethiopia to meet the Sustainable Development Goal (SDG) 3.6 target of reducing the mortality by 50% for the year 2020 (United Nations Sustainable Development Goals 2019). The 10-year trend of incidence, prevalence, DALYs and mortality from road traffic injuries in Ethiopia is also similar with that of Kenya, Tanzania, Uganda and Zambia (GBD 2017e).

The findings from this study show that in the past 10 years there is a decreasing trend in the number of deaths from injuries in Ethiopia. In comparison with Kenya, Uganda and Tanzania the magnitude and trend for the number of deaths observed in Ethiopia is consistent (GBD 2017f). However, as compared with Tanzania the number of deaths observed is higher. This could be attributed to the difference in coverage and quality of road infrastructure as well as legislative and regulatory efforts that might exist between eastern sub-Saharan African countries. Above all, the current number of deaths observed in eastern sub-Saharan Africa and Ethiopia is unacceptably high given that most injury deaths are preventable with moderate efforts.

One of the limitations of this study is associated with data sources. GBD has used 463 different data sources for all components of disease burden in Ethiopia. Seventy-three data sources are used to estimate the burden of injuries in Ethiopia, and the Addis Ababa Mortality Surveillance data, verbal autopsy data and demographic surveys conducted in Ethiopia are relied upon heavily (please see annex I for more detail). As a database study, some of the results might be modeled results depending on the data source availability. Therefore, some of the estimates could have a wide uncertainty interval, be lacking in precision, and/or underestimated. In spite of this, the paper presents the burden of injuries as the first national estimate for Ethiopia and underlines the need to establish a national system to capture all data on different injuries. We hope the output from this paper will have valuable input to influence policy for prospective national injury control efforts in Ethiopia.

## Conclusion and recommendations

In Ethiopia, deaths due to injuries are a growing public health problem. More than three-fourths of injury related deaths result from road injury, falls, self-harm and violence. The age-standardized death rate due to injuries is high and the current annual reduction is not satisfactory.

The current strategies in place to address the public health impact of road injuries are not sufficient to bring a marked reduction to the burden of road injuries. It calls for intensification of the efforts to enforce road safety laws and implement available legislative policies and guidelines efficiently to ensure safety. Furthermore, formulating new inclusive legislation, policies, guidelines and national strategies that address other forms of injuries would help to reduce the observed mortality.

The age standardized death rate due to self-harm in Ethiopia is high and increasing. This calls the country to consider self-harm as one of the public health priority problems.

Males, children under 5, children and young adults aged 15–24 years are highly affected by injuries. Children under 5 are particularly affected by drowning, falls and mechanical forces, while young adults are highly affected by road injuries. Therefore, any future efforts to reduce the burden of injury in Ethiopia should consider these sex, age and type of injury differences for better, more effective results. Finally, further study to estimate the burden of unintentional and intentional injuries is needed to understand the problem with better precision.

## Supplementary Information

**Additional file 1.** List of GBD data sources.

**Additional file 2.** Age standardized DALYs and Deaths resulted from injuries stratified by injury types among east African countries in 2007 and 2017.

**Additional file 3.** Number of deaths resulted from injuries stratified by injury types and age in 2007 and 2017.

## Data Availability

All data related with this manuscript is available at http://ghdx.healthdata.org/.

## Abbreviations

CODEm: Cause of Death Ensemble model
CSA: Central Statistics Agency
DALYs: Disability-Adjusted Life Years
EPHI: Ethiopian Public Health Institute
GBD: The Global Burden of Disease
GTP II: Growth and Transformation Plan two
HDSS: Health and Demographic Surveillance Sites
ICD: International Classification of Diseases
IHME: Institute for Health Metrics and Evaluation
NRTSC: National Road Traffic Safety Council
UI: Uncertainty Interval
UN SDG: United Nations Sustainable Development Goals
YLD: Years of Life Lived with Disability
YLL: Years of Life Lost
