# Supplementary material for: The burden of injuries in Ethiopia from 1990-2017: evidence from the global burden of disease study
Source: Inj Epidemiol. 2020 Dec 21;7:67. doi: 10.1186/s40621-020-00292-9 (PMC7751094; doi:10.1186/s40621-020-00292-9)
Supplement: Supplementary file 1 — Additional file 1. List of GBD data sources. [file 40621_2020_292_MOESM1_ESM.docx]

Additional file I GBD data sources used to estimate the burden of injuries in Ethiopian

| No. | Data sources | Data Collection Method | Year Start | Representativeness | Owner |
| --- | --- | --- | --- | --- | --- |
| 1 | Addis Ababa Mortality Surveillance Program 2006 | Verbal Autopsy | 2006 | Nationally representative only | Addis Ababa University. Ethiopia |
| 2 | Addis Ababa Mortality Surveillance Program 2007 | Verbal Autopsy | 2007 | Nationally representative only | Addis Ababa University. Ethiopia |
| 3 | Addis Ababa Mortality Surveillance Program 2008 | Verbal Autopsy | 2008 | Nationally representative only | Addis Ababa University. Ethiopia |
| 4 | Addis Ababa Mortality Surveillance Program 2009 | Verbal Autopsy | 2009 | Nationally representative only | Addis Ababa University. Ethiopia |
| 5 | Addis Ababa Mortality Surveillance Program 2010 | Verbal Autopsy | 2010 | Nationally representative only | Addis Ababa University. Ethiopia |
| 6 | Addis Ababa Mortality Surveillance Program 2011 | Verbal Autopsy | 2011 | Nationally representative only | Addis Ababa University. Ethiopia |
| 7 | Addis Ababa Mortality Surveillance Program 2012 | Verbal Autopsy | 2012 | Nationally representative only | Addis Ababa University. Ethiopia |
| 8 | Addis Ababa Mortality Surveillance Program 2015 | Verbal Autopsy | 2015 | Nationally representative only | Addis Ababa University. Ethiopia |
| 9 | Assessment of the Cause of Death and Characteristics of Chronic Illnesses in Addis Ababa with Emphasis on HIV/AIDS | Verbal Autopsy | 2003 | Unknown | Belete H. (A Community Based Study) [thesis]. Addis Ababa, Ethiopia: Addis Ababa University, 2005 |
| 10 | Battle Deaths Dataset Version 3.1, 2009 |  | 1980 |  | Peace Research Institute Oslo (PRIO), Oslo, Norway |
| 11 | Battle Deaths Dataset Version 3.1, 2009 |  | 1981 |  | Peace Research Institute Oslo (PRIO), Oslo, Norway |
| 12 | Battle Deaths Dataset Version 3.1, 2009 |  | 1982 |  | Peace Research Institute Oslo (PRIO), Oslo, Norway |
| 13 | Battle Deaths Dataset Version 3.1, 2009 |  | 1983 |  | Peace Research Institute Oslo (PRIO), Oslo, Norway |
| 14 | Battle Deaths Dataset Version 3.1, 2009 |  | 1984 |  | Peace Research Institute Oslo (PRIO), Oslo, Norway |
| 15 | Battle Deaths Dataset Version 3.1, 2009 |  | 1985 |  | Peace Research Institute Oslo (PRIO), Oslo, Norway |
| 16 | Battle Deaths Dataset Version 3.1, 2009 |  | 1986 |  | Peace Research Institute Oslo (PRIO), Oslo, Norway |
| 17 | Battle Deaths Dataset Version 3.1, 2009 |  | 1987 |  | Peace Research Institute Oslo (PRIO), Oslo, Norway |
| 18 | Battle Deaths Dataset Version 3.1, 2009 |  | 1988 |  | Peace Research Institute Oslo (PRIO), Oslo, Norway |
| 19 | Causes of Death among Children Aged 5 to 14 Years Old from 2008 to 2013 in Kersa Health and Demographic Surveillance System (Kersa HDSS), Ethiopia. | Verbal Autopsy | 2008 | Unknown | Dedefo M, Zelalem D, Eskinder B, Assefa N, Ashenafi W, Baraki N, Damena Tesfatsion M, Oljira L, Haile A. PLoS One. 2016; 11(6): e0151929 |
| 20 | Cause-Specific Mortality - Release 2014. Accra, Ghana: INDEPTH, 2014 | Verbal Autopsy | 1992 | Unknown | INDEPTH. Africa, Asia, Oceania - INDEPTH Network |
| 21 | Cause-Specific Mortality - Release 2014. Accra, Ghana: INDEPTH, 2014 | Verbal Autopsy | 1993 | Unknown | INDEPTH. Africa, Asia, Oceania - INDEPTH Network |
| 22 | Cause-Specific Mortality - Release 2014. Accra, Ghana: INDEPTH, 2014 | Verbal Autopsy | 1994 | Unknown | INDEPTH. Africa, Asia, Oceania - INDEPTH Network |
| 23 | Cause-Specific Mortality - Release 2014. Accra, Ghana: INDEPTH, 2014 | Verbal Autopsy | 1995 | Unknown | INDEPTH. Africa, Asia, Oceania - INDEPTH Network |
| 24 | Cause-Specific Mortality - Release 2014. Accra, Ghana: INDEPTH, 2014 | Verbal Autopsy | 1996 | Unknown | INDEPTH. Africa, Asia, Oceania - INDEPTH Network |
| 25 | Cause-Specific Mortality - Release 2014. Accra, Ghana: INDEPTH, 2014 | Verbal Autopsy | 1997 | Unknown | INDEPTH. Africa, Asia, Oceania - INDEPTH Network |
| 26 | Cause-Specific Mortality - Release 2014. Accra, Ghana: INDEPTH, 2014 | Verbal Autopsy | 1998 | Unknown | INDEPTH. Africa, Asia, Oceania - INDEPTH Network |
| 27 | Cause-Specific Mortality - Release 2014. Accra, Ghana: INDEPTH, 2014 | Verbal Autopsy | 1999 | Unknown | INDEPTH. Africa, Asia, Oceania - INDEPTH Network |
| 28 | Cause-Specific Mortality - Release 2014. Accra, Ghana: INDEPTH, 2014 | Verbal Autopsy | 2000 | Unknown | INDEPTH. Africa, Asia, Oceania - INDEPTH Network |
| 29 | Cause-Specific Mortality - Release 2014. Accra, Ghana: INDEPTH, 2014 | Verbal Autopsy | 2001 | Unknown | INDEPTH. Africa, Asia, Oceania - INDEPTH Network |
| 30 | Cause-Specific Mortality - Release 2014. Accra, Ghana: INDEPTH, 2014 | Verbal Autopsy | 2002 | Unknown | INDEPTH. Africa, Asia, Oceania - INDEPTH Network |
| 31 | Cause-Specific Mortality - Release 2014. Accra, Ghana: INDEPTH, 2014 | Verbal Autopsy | 2003 | Unknown | INDEPTH. Africa, Asia, Oceania - INDEPTH Network |
| 32 | Cause-Specific Mortality - Release 2014. Accra, Ghana: INDEPTH, 2014 | Verbal Autopsy | 2004 | Unknown | INDEPTH. Africa, Asia, Oceania - INDEPTH Network |
| 33 | Cause-Specific Mortality - Release 2014. Accra, Ghana: INDEPTH, 2014 | Verbal Autopsy | 2005 | Unknown | INDEPTH. Africa, Asia, Oceania - INDEPTH Network |
| 34 | Cause-Specific Mortality - Release 2014. Accra, Ghana: INDEPTH, 2014 | Verbal Autopsy | 2006 | Unknown | INDEPTH. Africa, Asia, Oceania - INDEPTH Network |
| 35 | Cause-Specific Mortality - Release 2014. Accra, Ghana: INDEPTH, 2014 | Verbal Autopsy | 2007 | Unknown | INDEPTH. Africa, Asia, Oceania - INDEPTH Network |
| 36 | Cause-Specific Mortality - Release 2014. Accra, Ghana: INDEPTH, 2014 | Verbal Autopsy | 2008 | Unknown | INDEPTH. Africa, Asia, Oceania - INDEPTH Network |
| 37 | Cause-Specific Mortality - Release 2014. Accra, Ghana: INDEPTH, 2014 | Verbal Autopsy | 2009 | Unknown | INDEPTH. Africa, Asia, Oceania - INDEPTH Network |
| 38 | Cause-Specific Mortality - Release 2014. Accra, Ghana: INDEPTH, 2014 | Verbal Autopsy | 2010 | Unknown | INDEPTH. Africa, Asia, Oceania - INDEPTH Network |
| 39 | Cause-Specific Mortality - Release 2014. Accra, Ghana: INDEPTH, 2014 | Verbal Autopsy | 2011 | Unknown | INDEPTH. Africa, Asia, Oceania - INDEPTH Network |
| 40 | Cause-Specific Mortality - Release 2014. Accra, Ghana: INDEPTH, 2014 | Verbal Autopsy | 2012 | Unknown | INDEPTH. Africa, Asia, Oceania - INDEPTH Network |
| 41 | Determinants of under-five mortality in Gilgel Gibe Field Research Center, Southwest Ethiopia. | Verbal Autopsy | 2004 | Unknown | Deribew A, Tessema F, Girma B. Ethiop J Health Dev. 2007; 21(2): 117-24 |
| 42 | Emerging chronic non-communicable diseases in rural communities of Northern Ethiopia: evidence using population-based verbal autopsy method in Kilite Awlaelo surveillance site. | Verbal Autopsy | 2010 | Unknown | Weldearegawi B, Ashebir Y, Gebeye E, Gebregziabiher T, Yohannes M, Mussa S, Berhe H, Abebe Z. Health Policy Plan. 2013. [Epub ahead of print] |
| 43 | Ethiopia Demographic Surveillance Verbal Autopsy Data 2009-2016 | Verbal Autopsy | 2009 | Unknown | Ethiopian Public Health Association. |
| 44 | Ethiopia Demographic Surveillance Verbal Autopsy Data 2009-2016 | Verbal Autopsy | 2010 | Unknown | Ethiopian Public Health Association. |
| 45 | Ethiopia Demographic Surveillance Verbal Autopsy Data 2009-2016 | Verbal Autopsy | 2011 | Unknown | Ethiopian Public Health Association. |
| 46 | Ethiopia Demographic Surveillance Verbal Autopsy Data 2009-2016 | Verbal Autopsy | 2012 | Unknown | Ethiopian Public Health Association. |
| 47 | Ethiopia Demographic Surveillance Verbal Autopsy Data 2009-2016 | Verbal Autopsy | 2013 | Unknown | Ethiopian Public Health Association. |
| 48 | Factors associated with place of death in Addis Ababa, Ethiopia. | Verbal Autopsy | 2008 | Unknown | Anteneh A, Araya T, Misganaw A. BMC Palliat Care. 2013; 12(14): 14 |
| 49 | Global Terrorism Database |  | 1984 |  | National Consortium for the Study of Terrorism and Responses to Terrorism (START), College Park , MD, United States of America: University of Maryland, 2018 |
| 50 | Global Terrorism Database |  | 1986 |  | National Consortium for the Study of Terrorism and Responses to Terrorism (START), College Park , MD, United States of America: University of Maryland, 2018 |
| 51 | Global Terrorism Database |  | 1989 |  | National Consortium for the Study of Terrorism and Responses to Terrorism (START), College Park , MD, United States of America: University of Maryland, 2018 |
| 52 | Global Terrorism Database |  | 1990 |  | National Consortium for the Study of Terrorism and Responses to Terrorism (START), College Park , MD, United States of America: University of Maryland, 2018 |
| 53 | Global Terrorism Database |  | 1991 |  | National Consortium for the Study of Terrorism and Responses to Terrorism (START), College Park , MD, United States of America: University of Maryland, 2018 |
| 54 | Global Terrorism Database |  | 1992 |  | National Consortium for the Study of Terrorism and Responses to Terrorism (START), College Park , MD, United States of America: University of Maryland, 2018 |
| 55 | Global Terrorism Database |  | 1994 |  | National Consortium for the Study of Terrorism and Responses to Terrorism (START), College Park , MD, United States of America: University of Maryland, 2018 |
| 56 | Global Terrorism Database |  | 1995 |  | National Consortium for the Study of Terrorism and Responses to Terrorism (START), College Park , MD, United States of America: University of Maryland, 2018 |
| 57 | Global Terrorism Database |  | 1996 |  | National Consortium for the Study of Terrorism and Responses to Terrorism (START), College Park , MD, United States of America: University of Maryland, 2018 |
| 58 | Global Terrorism Database |  | 2000 |  | National Consortium for the Study of Terrorism and Responses to Terrorism (START), College Park , MD, United States of America: University of Maryland, 2018 |
| 59 | Global Terrorism Database |  | 2005 |  | National Consortium for the Study of Terrorism and Responses to Terrorism (START), College Park , MD, United States of America: University of Maryland, 2018 |
| 60 | Global Terrorism Database |  | 2006 |  | National Consortium for the Study of Terrorism and Responses to Terrorism (START), College Park , MD, United States of America: University of Maryland, 2018 |
| 61 | Global Terrorism Database |  | 2008 |  | National Consortium for the Study of Terrorism and Responses to Terrorism (START), College Park , MD, United States of America: University of Maryland, 2018 |
| 62 | Global Terrorism Database |  | 2011 |  | National Consortium for the Study of Terrorism and Responses to Terrorism (START), College Park , MD, United States of America: University of Maryland, 2018 |
| 63 | Global Terrorism Database |  | 2013 |  | National Consortium for the Study of Terrorism and Responses to Terrorism (START), College Park , MD, United States of America: University of Maryland, 2018 |
| 64 | Global Terrorism Database |  | 2015 |  | National Consortium for the Study of Terrorism and Responses to Terrorism (START), College Park , MD, United States of America: University of Maryland, 2018 |
| 65 | Global Terrorism Database |  | 2016 |  | National Consortium for the Study of Terrorism and Responses to Terrorism (START), College Park , MD, United States of America: University of Maryland, 2018 |
| 66 | HIV/AIDS-Related Mortality In Addis Ababa City Administration [thesis]. | Verbal Autopsy | 2001 | Unknown | Araya T. Addis Ababa, Ethiopia: Addis Ababa University, 2001 |
| 67 | La vigilancia de entierros detectÃ³ una reducciÃ³n significativa en las muertes relacionadas con el VIH en Addis Ababa, EtiopÃ­a. | Verbal Autopsy | 2001 | Unknown | Araya T, Tensou B, Davey G, Berhane Y. Trop Med Int Health. 2011; 16(12): 1483-9 |
| 68 | La vigilancia de entierros detectÃ³ una reducciÃ³n significativa en las muertes relacionadas con el VIH en Addis Ababa, EtiopÃ­a. | Verbal Autopsy | 2003 | Unknown | Araya T, Tensou B, Davey G, Berhane Y. Trop Med Int Health. 2011; 16(12): 1483-9 |
| 69 | Maternal mortality in Addis Ababa, Ethiopia. | Survey/Census | 1982 | Unknown | Kwast BE, Rochat RW, Kidane-Mariam W. Stud Fam Plann. 1986; 17(6 (Pt 1)): 288-301 |
| 70 | Patterns of childhood mortality in three districts of north Gondar Administrative Zone. A community based study using the verbal autopsy method. | Verbal Autopsy | 1992 | Unknown | Fantahun M. Ethiop Med J. 1998; 36(2): 71-81 |
| 71 | Perinatal Mortality Magnitude, Determinants and Causes in West Gojam: Population-Based Nested Case-Control Study. | Verbal Autopsy | 2011 | Unknown | Yirgu R, Molla M, Sibley L, Gebremariam A. PLoS One. 2016; 11(7): e0159390 |
| 72 | The Butajira rural health project in Ethiopia: mortality pattern of the under fives. | Verbal Autopsy | 1987 | Unknown | Shamebo D, Muhe L, SandstrÃ¶m A, Wall S. : mortality pattern of the under fi |
| 73 | The use of simplified verbal autopsy in identifying causes of adult death in a predominantly rural population in Ethiopia. | Verbal Autopsy | 1997 | Unknown | Lulu K, Berhane Y. BMC Public Health. 2005; 5: 58 |

Source by injury type

| Source of data/Injury Type | Injuries | Animal contact | Conflict and terrorism | Drowning | Environmental heat and cold exposure | Executions and police conflict | Exposure to forces of nature | Exposure to mechanical forces | Falls | Fire, heat, and hot substances | Foreign body | Interpersonal violence | Other exposure to mechanical forces | Other unintentional injuries | Physical violence by firearm | Physical violence by other means | Physical violence by sharp object | Poisoning by gas | Poisonings | Self-harm | Self-harm and interpersonal violence | Transport injuries | Unintentional firearm injuries | Unintentional injuries | Venomous animal contact |
| --- | --- | --- | --- | --- | --- | --- | --- | --- | --- | --- | --- | --- | --- | --- | --- | --- | --- | --- | --- | --- | --- | --- | --- | --- | --- |
| Addis Ababa Mortality Surveillance Program | X | X | X | X | X |  | X | X | X | X | X | X | X | X | X | X | X | X | X | X | X | X | X | X | X |
| Anteneh A, Araya T, Misganaw A. Factors associated with place of death in Addis Ababa, Ethiopia. BMC Palliat Care. 2013; 12(14): 14 | X |  |  |  |  |  |  |  |  |  |  |  |  |  |  |  |  |  |  |  |  |  |  |  |  |
| Araya T, Tensou B, Davey G, Berhane Y. La vigilancia de entierros detectÃ³ una reducciÃ³n significativa en las muertes relacionadas con el VIH en Addis Ababa, EtiopÃ­a. Trop Med Int Health. 2011; 16(12): 1483-9 | X |  |  |  |  |  |  |  |  |  |  |  |  |  |  |  |  |  |  |  | X | X |  |  |  |
| Araya T. HIV/AIDS-Related Mortality In Addis Ababa City Administration [thesis]. Addis Ababa, Ethiopia: Addis Ababa University, 2001 | X |  |  |  |  |  |  |  |  |  |  |  |  |  |  |  |  |  |  |  |  |  |  |  |  |
| Belete H. Assessment of the Cause of Death and Characteristics of Chronic Illnesses in Addis Ababa with Emphasis on HIV/AIDS (A Community Based Study) [thesis]. Addis Ababa, Ethiopia: Addis Ababa University, 2005 | X |  |  |  |  |  |  | X | X |  |  |  |  |  |  |  |  |  |  | X | X |  | X | X |  |
| Dedefo M, Zelalem D, Eskinder B, Assefa N, Ashenafi W, Baraki N, Damena Tesfatsion M, Oljira L, Haile A. Causes of Death among Children Aged 5 to 14 Years Old from 2008 to 2013 in Kersa Health and Demographic Surveillance System (Kersa HDSS), Ethiopia. PLoS One. 2016; 11(6): e0151929 | X |  |  | X |  |  |  |  |  | X |  |  |  |  |  |  |  |  |  |  |  |  |  | X |  |
| Deribew A, Tessema F, Girma B. Determinants of under-five mortality in Gilgel Gibe Field Research Center, Southwest Ethiopia. Ethiop J Health Dev. 2007; 21(2): 117-24 | X |  |  |  |  |  |  |  |  |  |  |  |  |  |  |  |  |  |  |  |  |  |  |  |  |
| Ethiopia Demographic Surveillance Verbal Autopsy Data 2009-2016 | X | X |  | X |  |  | X |  | X |  |  | X |  |  |  |  |  |  | X | X | X | X |  | X |  |
| Fantahun M. Patterns of childhood mortality in three districts of north Gondar Administrative Zone. A community based study using the verbal autopsy method. Ethiop Med J. 1998; 36(2): 71-81 | X | X |  | X |  |  |  | X |  |  |  |  | X |  |  |  |  |  |  |  |  |  |  | X | X |
| INDEPTH. Africa, Asia, Oceania - INDEPTH Network Cause-Specific Mortality - Release 2014. Accra, Ghana: INDEPTH, 2014 | X |  |  | X |  |  |  |  | X | X |  | X |  |  |  |  |  |  | X | X | X | X |  | X | X |
| Kwast BE, Rochat RW, Kidane-Mariam W. Maternal mortality in Addis Ababa, Ethiopia. Stud Fam Plann. 1986; 17(6 (Pt 1)): 288-301 | X |  |  |  |  |  |  |  |  |  |  |  |  |  |  |  |  |  |  |  |  |  |  |  |  |
| Lulu K, Berhane Y. The use of simplified verbal autopsy in identifying causes of adult death in a predominantly rural population in Ethiopia. BMC Public Health. 2005; 5: 58 | X |  |  |  |  |  |  |  |  |  |  |  |  |  |  |  |  |  |  |  |  |  |  |  |  |
| National Consortium for the Study of Terrorism and Responses to Terrorism (START). Global Terrorism Database. College Park , MD, United States of America: University of Maryland, 2018 | X |  | X |  |  |  |  |  |  |  |  |  |  |  |  |  |  |  |  |  |  |  |  |  |  |
| Peace Research Institute Oslo (PRIO). Battle Deaths Dataset Version 3.1, 2009. Oslo, Norway: Peace Research Institute Oslo (PRIO), 2009 | X |  | X |  |  | X |  |  |  |  |  |  |  |  |  |  |  |  |  |  |  |  |  |  |  |
| Shamebo D, Muhe L, SandstrÃ¶m A, Wall S. The Butajira rural health project in Ethiopia: mortality pattern of the under fives. J Trop Pediatr. 1991; 37(5): 254-61 | X |  |  |  |  |  |  |  |  |  |  |  |  |  |  |  |  |  |  |  |  |  |  |  |  |
| Weldearegawi B, Ashebir Y, Gebeye E, Gebregziabiher T, Yohannes M, Mussa S, Berhe H, Abebe Z. Emerging chronic non-communicable diseases in rural communities of Northern Ethiopia: evidence using population-based verbal autopsy method in Kilite Awlaelo surveillance site. Health Policy Plan. 2013. [Epub ahead of print] | X |  |  | X |  |  | X |  | X |  |  | X |  |  |  |  |  |  |  | X | X |  |  | X |  |
| Yirgu R, Molla M, Sibley L, Gebremariam A. Perinatal Mortality Magnitude, Determinants and Causes in West Gojam: Population-Based Nested Case-Control Study. PLoS One. 2016; 11(7): e0159390 | X |  |  |  |  |  |  |  | X |  | X |  |  |  |  |  |  |  |  |  |  |  |  | X |  |
