# Supplementary material for: The burden of injuries in Ethiopia from 1990-2017: evidence from the global burden of disease study
Source: Inj Epidemiol. 2020 Dec 21;7:67. doi: 10.1186/s40621-020-00292-9 (PMC7751094; doi:10.1186/s40621-020-00292-9)

Additional file II Number of deaths resulted from injuries stratified by injury types and age in 2007 and 2017


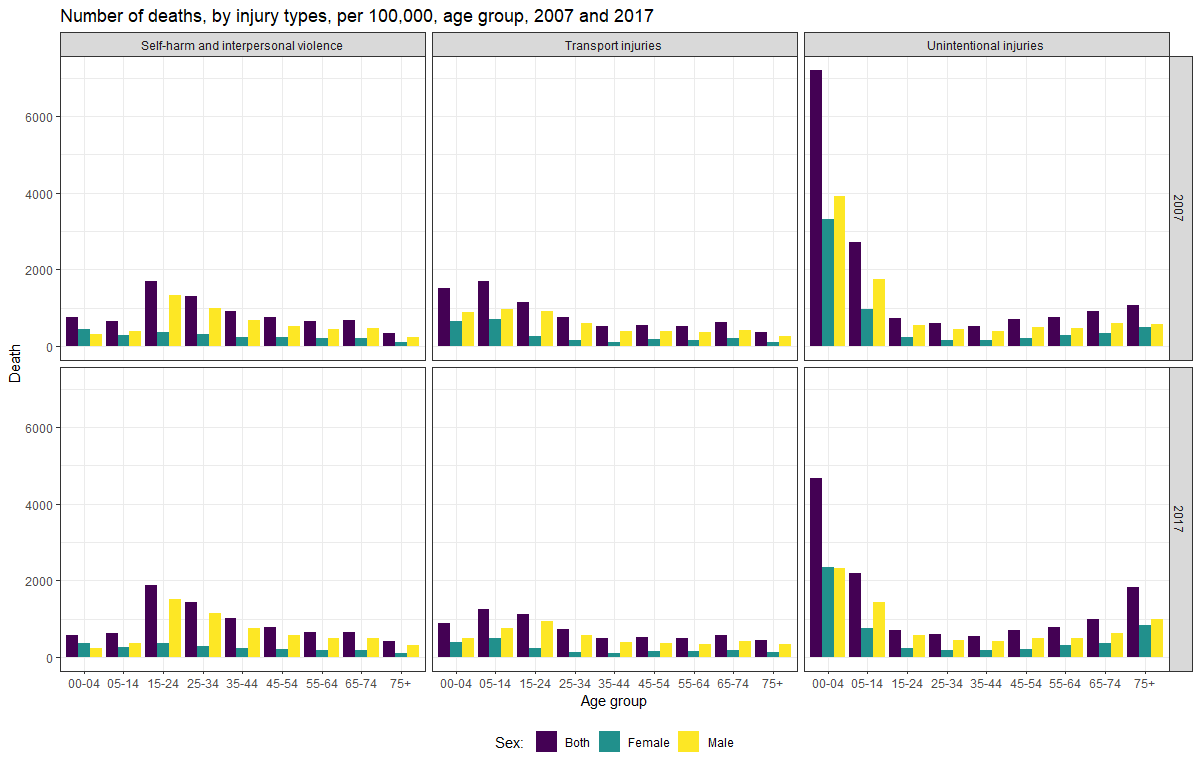

Supplement: Supplementary file 2 — Additional file 2. Age standardized DALYs and Deaths resulted from injuries stratified by injury types among east African countries in 2007 and 2017. [file 40621_2020_292_MOESM2_ESM.docx]
