# Supplementary material for: The burden of injuries in Ethiopia from 1990-2017: evidence from the global burden of disease study
Source: Inj Epidemiol. 2020 Dec 21;7:67. doi: 10.1186/s40621-020-00292-9 (PMC7751094; doi:10.1186/s40621-020-00292-9)

Additional file III Age standardized DALYs and Deaths resulted from injuries stratified by injury types among east African countries in 2007 and 2017


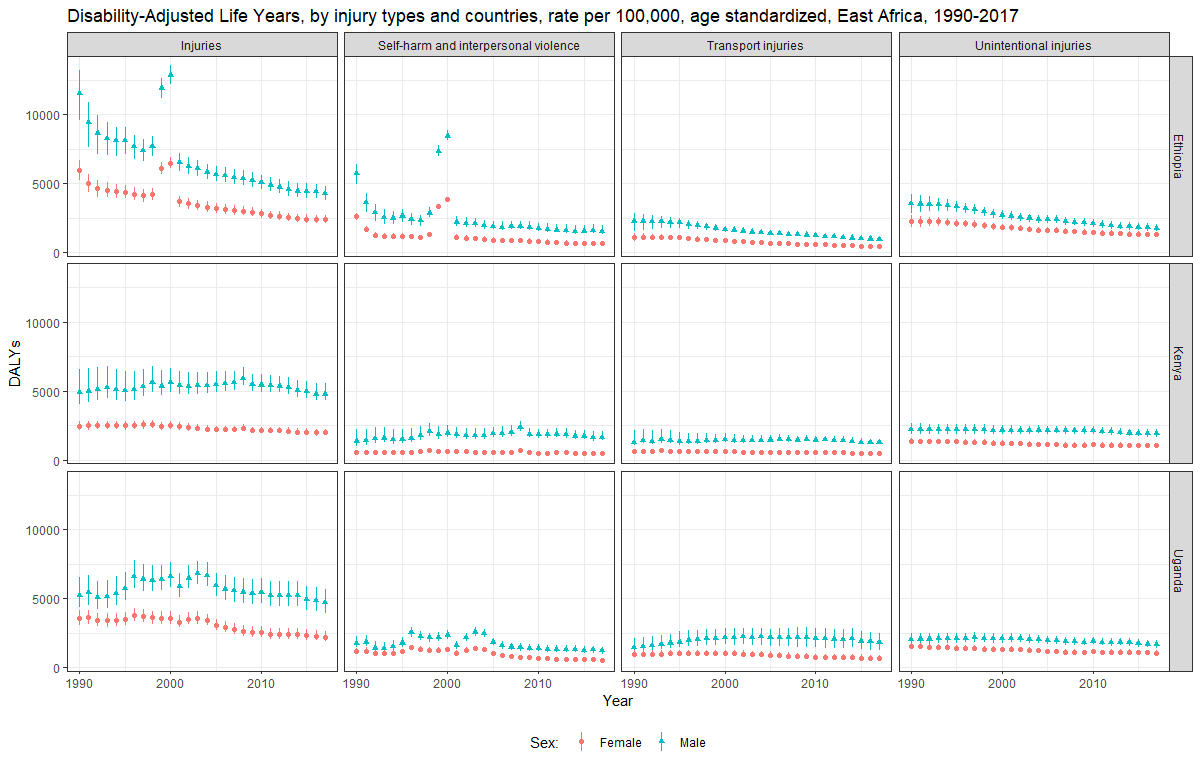


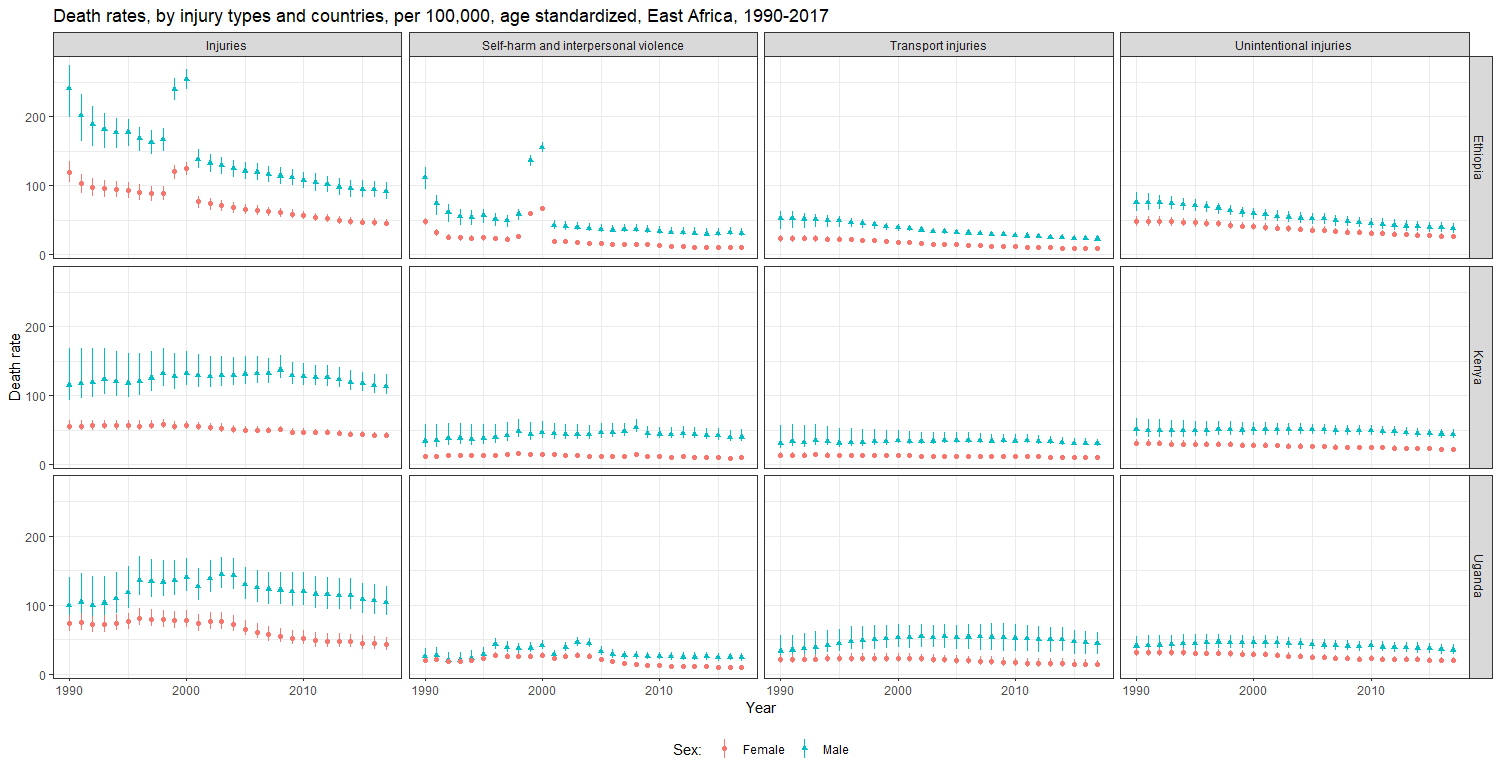

Supplement: Supplementary file 3 — Additional file 3. Number of deaths resulted from injuries stratified by injury types and age in 2007 and 2017. [file 40621_2020_292_MOESM3_ESM.docx]
